# An Air Velocity Monitor for Coal Mine Ventilation Based on Vortex-Induced Triboelectric Nanogenerator

**DOI:** 10.3390/s22134832

**Published:** 2022-06-26

**Authors:** Guocheng Shen, Jijie Ma, Yili Hu, Jianping Li, Tinghai Cheng, Jianming Wen

**Affiliations:** 1 The Institute of Precision Machinery and Smart Structure, College of Engineering, Zhejiang Normal University, Yinbin Street 688, Jinhua 321004, China; shenguocheng@zjnu.edu.cn (G.S.); mjj@zjnu.cn (J.M.); huyili@zjnu.edu.cn (Y.H.); lijp@zjnu.cn (J.L.); 2 Key Laboratory of Intelligent Operation and Maintenance Technology & Equipment for Urban Rail Transit of Zhejiang Province, Zhejiang Normal University, Yinbin Street 688, Jinhua 321004, China; 3 Beijing Institute of Nanoenergy and Nanosystems, Chinese Academy of Sciences, Beijing 101400, China

**Keywords:** vortex-induced vibration, vortex-induced triboelectric nanogenerator, air velocity monitor, ventilation

## Abstract

Air velocity of coal mine ventilation is an important consideration that may cause serious damage. This paper proposes a simple, low cost and effective air velocity monitor (AVM) for coal mine ventilation. The AVM uses the lock-in characteristic of vortex-induced vibration (VIV) to sense the air velocity. Amplitude of the VIV is converted into frequency signal of a vortex-induced triboelectric nanogenerator (VI-TENG) to improve the durability. Structure of the AVM are designed, and parameters of the AVM are optimized with experiments. For the upper and lower air velocity thresholds of 3.1 and 3.6 m/s, the optimized flexible beam length, slider weight, electrode space and electrode width are 42.5 mm, 0.4 g, 0.2 mm and 0.5 mm, respectively. Experiments also show that the output frequency of the VI-TENG could represent the amplitude of VIV well with the correlation coefficient of 0.93. Durability test demonstrates that the AVM generates stable output frequency in 120,000 cycles. A prototype and its controller are fabricated. Wind tunnel tests of this prototype show that it can give alarm when the gas velocity goes above the upper threshold or below the lower threshold. The proposed AVM could be a good solution for simple and effective coal mine ventilation alarm.

## 1. Introduction

Ventilation is an important safety configuration for underground coal mines. It significantly affects the behavior of methane layering and dispersion of methane which may lead to an explosion hazard [1]. The dust, CO and CO_2_ concentrations are also affected by ventilation [2,3]. As one of the key parameters of coal mine ventilation, air velocity (or air flowrate) is usually set to certain range to ensure the effectiveness of ventilation [1,4]. Common ways of monitoring air velocity involve detecting the air velocity with sensors, such as vane flow meter, hot-wire anemometer and Pitot tube flow meter. Vane flow meter is a popular flow meter thanks to the feature of low cost and high durability [5,6]. By counting the number of rotations to measure air velocity, vane has quite good anti-interference behavior. However, the accuracy and resistance to the airflow limits its application in demanding occasions. Hot-wire anemometer works on the principle of Joule heating and the consequent change of resistance [7]. It has good accuracy and less impact on the flow. However, in the dusty and flammable atmosphere of coal mines, it may lead to safety problems. Pitot tube detects air flow/velocity by the air pressure difference between the total pressure and static pressure which is characterized by the hydraulic height [8,9]. It requires an external sensor to detect the hydraulic height, and the air cleanliness is demanding. In the meantime, using anemometers to monitor air velocity needs an upstream computer to process the data of sensor, adding cost and complexity to the monitoring system. A special purpose, integrated and low-cost air velocity monitor is still in need for coal mine ventilation.

The air velocity of the ventilation system is set to a certain range, and the monitor gives alarm when the air velocity goes beyond the range. A direct way of air velocity monitor is to find a physical quantity with the same trend, i.e., works normally within the range and stops if it goes above the upper threshold or below the lower threshold. As a basic form of vibration in the fluid environment, vortex-induced vibration (VIV) can be used to measure the flow velocity of the fluid. The method is to set a fixed obstacle (bluff body) in the path of the flowing fluid and measures the frequency of vortices that arise behind the bluff body to calculate the flow velocity. However, this method has a high requirement on the accuracy of measuring instruments and is easily disturbed by environmental noise [10,11]. If the bluff body is connected to an elastic body, the movement will show the characteristic of lock-in which features an almost constant vibration frequency but parabola-like amplitude as the flow velocity increases [12,13,14,15,16]. This matches the requirement of air velocity monitor well. If the amplitudes of the VIV at the upper and lower threshold of the air velocity are equivalent, the amplitude will be the only parameter for alarm judgement. Amplitudes of VIV higher than a threshold represents normal air velocity and vice versa. However, detection of amplitudes involves fast response sensor or solving complicated mechanic field, an approach provides an estimate by relating the power in the radiation resistance of the transducer’s equivalent circuit to the acoustic radiation into the liquid [17], which is complex and uneconomical. An alternative is to use triboelectric nanogenerator (TENG) proposed by Wang [18,19,20]. Based on the Maxwell displacement current theory, TENG has wide selection of materials, demonstrating outstanding characteristics of low cost and simple fabrication [21,22,23]. In recent years, TENG have been proven to obtain energy from various environments such as wind, water wave, human motion and mechanical vibration through experimental and theoretical research [24,25,26,27,28,29]. The output of the TENG has strong regularity and stability, so it has been applied to various generators and sensors [30,31,32]. A sweep-type triboelectric linear motion sensor represents displacement with the frequency of signals from a group of staggered electrodes [33]. It shows a durable behavior, and could be an effective solution of amplitude detection.

This study proposes a special purpose air velocity monitor for coal mine ventilation based on VIV and VI-TENG. A bluff body is adopted to generate VIV as ventilation air flows and a VI-TENG represents the amplitude of VIV with frequency of output signals. The air velocity of ventilation could be monitored by simply monitoring the output frequency of VI-TENG, which ensures the special purpose, low cost and durable design. The destination air velocity range is selected according to the ventilation air velocity requirements of a coal mine with the lower and upper thresholds of 3.1 and 3.6 m/s, respectively. The structure, working principle of the monitor and experiments are provided. The results show that the monitor can generate alarm as required and the output frequency keep stable in 120,000 cycles.

## 2. Materials and Methods

### 2.1. Structural Design and Working Principle

**Figure 1:**
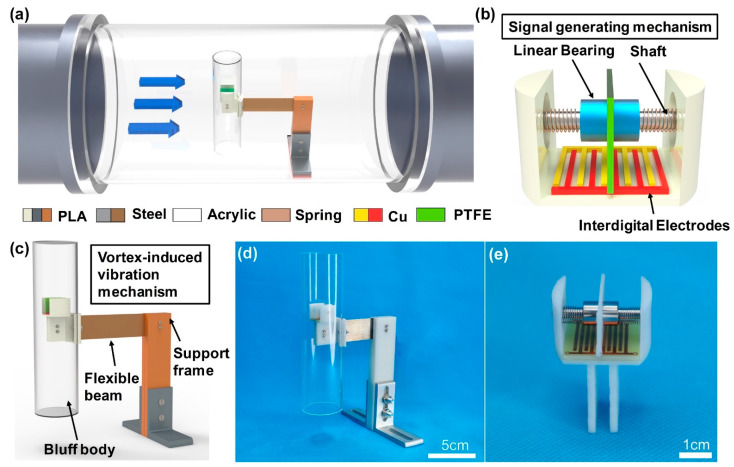
Schematic diagrams of the AVM: (**a**) the AVM in pipeline; (**b**) the structure of the signal generating mechanism; (**c**) schematic diagram of AVM; (**d**,**e**) prototypes of the AVM.

The system block diagram and structural design of the AVM are shown in Figure 1. The AVM consists of a vortex-induced vibration mechanism and a signal generating mechanism. The vortex-induced vibration mechanism aims to generate vibration with the help of the pipeline air flow. When fluid passes around a slender structure (cylindrical bluff body), the bluff body vibrates, which is caused by aerodynamic instability or vortex shedding. Therefore, the vortex-induced vibration mechanism composed of a flexible beam, support and cylindrical bluff body was designed, as shown in Figure 1c. Considering the size of signal generating mechanism and the aspect ratio of the bluff body, a cylindrical bluff body with diameter of 45 mm and a length of 170 mm was selected. The tail of the flexible beam made of steel is connected to the fixed end through two symmetrical support plates, and the front end is connected with a hollow cylindrical shell with length 170 mm and outer diameter 45 mm. The signal generating mechanism (Figure 1b) is installed inside the cylindrical bluff body. It is composed of two interdigital electrodes, a PTFE block, two springs, a linear bearing, a shaft and a base. The PTFE block moves along with the linear bearing, sliding against the interdigital electrodes to generate pulses. The springs and slider (the PTFE block and linear bearing) make up a vibration system that smooths the sliding process. The pre-tightening force between the slider and the interdigital electrodes could be regulated by adjusting the distance between the shaft and electrodes. The prototype of the AVM is illustrated in Figure 1d,e.

### 2.2. Working Principle

**Figure 2:**
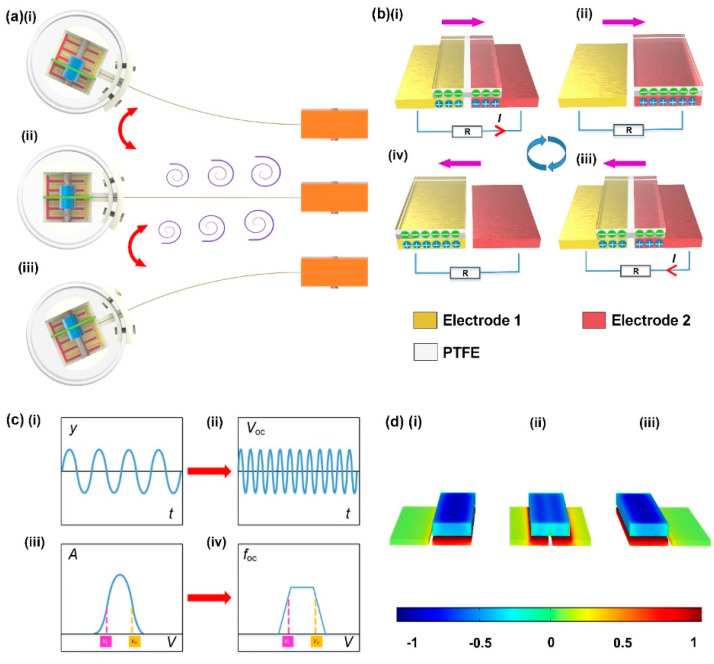
The working mechanism of AVM: (**a**) vortex vibration states of AVM; (**b**) illustration of the working principle of VI−TENG; (**c**) theoretical relationship between vibration signal and electrical signal of AVM: (i) displacement, (ii) open circuit voltage, (iii) amplitude, (iv) frequency; (**d**) simulating potentials of the VI−TENG in three different states simulated using COMSOL: (i) the state of alignment between the PTFE film and electrode−1, (ii) the state when the PTFE film is across the two electrodes, (iii) the state of alignment between the PTFE film and electrode−2.

The working process of the AVM is shown in Figure 2. When the gas bypasses the cylindrical bluff body, two rows of vortex with opposite rotation directions fall off both sides of the bluff body. The pressure of the asymmetric periodic flow field produced by the vortex rows forces the bluff body and the flexible beam vibrate. The vibration will apply periodic excitation to the slider via the springs, leading to the relative sliding between the slider and electrodes. Since the contact pairs, the PTFE block on the slider and the copper electrodes, have quite different friction polarities, the sliding will generate charge transfer between the two materials. When the slider is in the position shown in Figure 2(bi), the surfaces of the two pairs of copper electrodes carry the same amount of positive charge. As the slider moves to the position shown in Figure 2(bii), positive charges flow from electrode-1 to electrode-2 in the sliding direction to balance the potential difference, thereby forming a transient current in the load. When the slider keeps on moving, the positive charge returns to copper-1 under electrostatic induction, and the reversed current is generated in the load. The reversed current ends in the aligned position of PTFE and electrode-1, and another cycle starts immediately. The potentials of PTFE and electrodes at different position are simulated in COMSOL Multiphysics software, as demonstrated in Figure 2d. The block on the top is a PTFE film, selected from the MEMS library of COMSOL. The material of the electrodes are copper, selected from the AC/DC library of COMSOL. Three representative contact states are defined, the state of alignment between the PTFE film and electrode-1 (Figure 2(di)), the state of alignment between the PTFE film and electrode-2 (Figure 2(di)) and the state when the PTFE film is across the two electrodes (Figure 2(dii)).

Since the vortex vibration is the lock-in region, the vibration frequency (*f*_v_) is approximately fixed, the theoretical relationship between the vibration signal and the electrical signal of the AVM is shown in Figure 2c. The output frequency of AVM is several times of *f*_v_, depending mainly on the number of electrode pairs that the PTFE slides over in a cycle, while the latter is decided by the amplitude of the vortex vibration. Hence, the output frequency is positively correlated with the amplitude of the vortex vibration. Consider the vortex vibration works in a fixed range of gas velocity, the output pulses will emerge in the same region. When the gas velocity goes beyond the working range defined by the upper and lower velocity (*V*_L_ and *V*_U_), the output frequency decreases along with the amplitude of vortex vibration, and the alarm signal could be generated by monitoring the output frequency. Due to the space of the signal generating mechanism, the travel of the slider is limited. The output frequency will get saturated at high amplitudes of the vortex-induced vibration. The alarm thresholds should be configured out of this range, as illustrated in Figure 2(ciii),(civ).

## 3. Results

### 3.1. Output Performance

#### 3.1.1. Optimization of Vortex Vibration Parameters

The AVM responds to the gas velocity via vortex vibration at certain range and transform the vibration into pulses with a triboelectric nanogenerator. Vortex vibration parameters are crucial to the performance of AVM. Diameter and length of the bluff body have been selected according to the rules in reference [13] and the pipeline dimensions. The length of the beam and the mass of the vibrator will be studied in this part. Since many parts of the vibrator, such as the bluff body, the electrodes, the shaft, have been selected, the most convenient way of adjusting the mass is changing the mass of the slider. Thus, the mass of the slider is used in the coming study.

**Figure 3:**
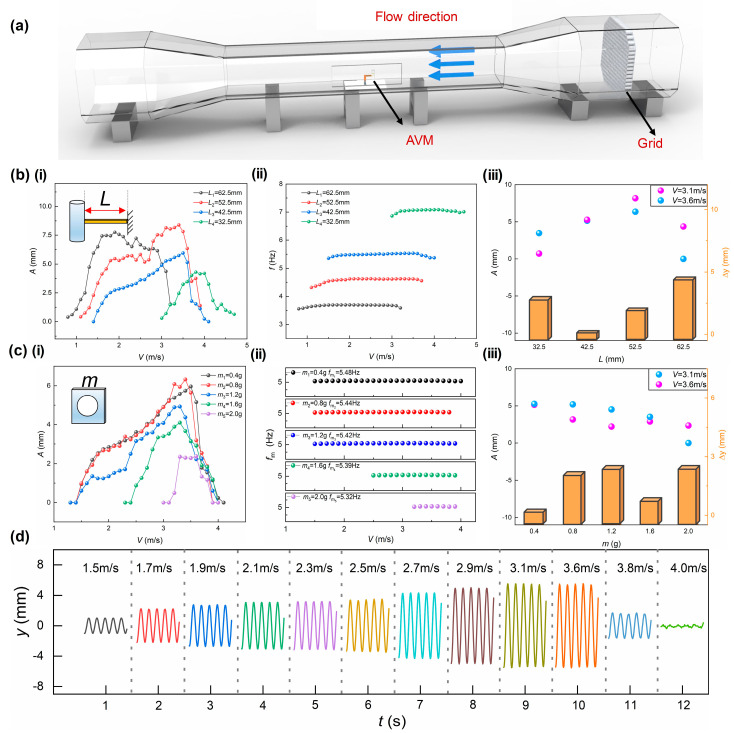
Optimization of vortex vibration parameters. (**a**) Schematic of the experimental apparatus; (**b**) vibration characteristics of AVM under beams with different length: (i) vibration amplitude, (ii) vibration frequency, (iii) D−value of amplitude; (**c**) vibration characteristics of AVM under different mass slider: (i) vibration amplitude, (ii) vibration frequency, (iii) D−value of amplitude; (**d**) real−time vibration signals of AVM at different wind speed.

The experiments are carried out in a wind tunnel as shown in Figure 3a. The AVM is placed in the tunnel and the gas velocity is adjusted with a frequency inverter. The length of the beam is increased by 10 mm, from 32.5 mm to 62.5 mm. As the gas velocity increases, the bluff body starts vibrating at an almost constant frequency, called lock-in in reference [34], while the amplitude of the vortex vibration (*A*) goes up to the peak and then goes down to zero gradually, as illustrated in Figure 3(bi),(bii). This is consistent with theory that when these vortices are shed at a frequency near one of the natural frequencies of the harvester, lock-in takes place and the amplitude of the device increases significantly. As the flow rate increases further, the device desynchronizes and the amplitude gradually decreases. In order to detecting the gas leakage, the AVM should generate the same amplitude of vibration at the upper and lower velocity threshold to benefit the judgement of alarm. In this study, the upper and lower threshold of the mine ventilation system are 3.6 and 3.1 m/s, respectively. Thus, the length of the beam is selected as 42.5 mm at which the amplitudes of vibration are both 5 mm for the upper and lower threshold.

Figure 3c illustrates the vibration behavior at different masses of the slider. The mass goes from 0.4 g, the minimum accessible mass of the slider, to 2.0 g increased by 0.4 g step. Above 2.0 g, the vortex vibration could not be induced in the common working range of velocity. It could be seen that the amplitude decreases dramatically and the frequency decreases slightly as the mass of the slider increases. The reason is that the interference of the internal vibration system to the external increases. Both 0.4 g and 1.6 g generate similar amplitudes of vibration at the upper and lower threshold, but the latter involves higher energy consumption and a consequent short service life. Then, the mass of the slider is selected as 0.4 g. Figure 3d demonstrates the real-time vibration signals at the selected parameters, 42.5 mm and 0.4 g. The amplitude of the vibration is the same at 3.1 and 3.6 m/s and begin to decrease when the gas velocity goes away from the working range, which provides the basis of gas leakage detection.

#### 3.1.2. Optimization of VI-TENG Parameters

Vortex-induced triboelectric nanogenerator is adopted to transform the amplitude of vortex vibration into pulse frequency. Generally, more pairs of electrodes in unit length will produce higher resolution of VI-TENG. However, the signal in this case is also vulnerable to interference due to the fade of amplitude. In this part, the width of electrode (*D*) and the space between electrodes (*d*) will be optimized to balance the resolution and amplitude. The length of beam and mass of slider use the optimized value above.

**Figure 4:**
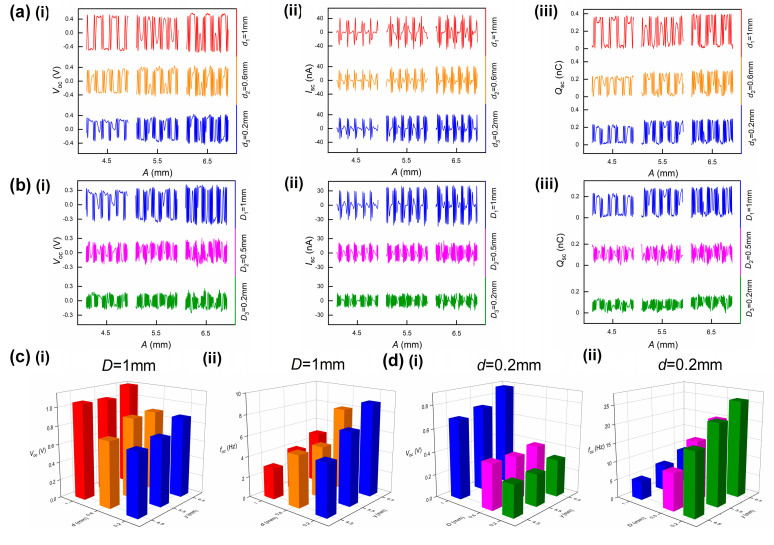
Optimization of electrodes’ width and spacing. (**a**) VI−TENG’s performance at different spacing: (i) open circuit voltage, (ii) short circuit current and (iii) amount of charge transferred; (**b**) VI−TENG’s performance at different widths: (i) open circuit voltage, (ii) short circuit current and (iii) amount of charge transferred; (**c**) characteristics of open circuit voltage at different spacing: (i) amplitude and (ii) frequency; (**d**) characteristics of open circuit voltage at different widths: (i) amplitude and (ii) frequency.

Three interdigital electrodes with spacing of 0.2, 0.6 and 1.0 mm and width of 1.0 mm are prepared for the spacing test. The AVM are fixed on a vibration exciter and excited at amplitudes of 4.5, 5.5 and 6.5 mm, covering the amplitudes of vortex vibration in the working velocity range. Specific details are shown in Figure S1 in the Support Information. The output performance of VI-TENG, the open circuit voltage (*V*_oc_), short circuit current (*I*_SC_) and amount of charge transferred (*Q*_SC_), are illustrated in Figure 4a,c. The spacing does not affect the performance of VI-TENG much, as the three performance indexes have slight changes for the three samples. The increase of excitation amplitude and decrease of spacing both benefit improving the frequency of VI-TENG since they both increase the number of electrode pairs covered by the slider in a cycle. Then the spacing between electrodes is selected as 0.2 mm which generates more pulses in a cycle.

Figure 4b,d shows the performance of VI-TENG at different widths of the electrode. With the decrease of the electrode width, the performance of VI-TENG goes down dramatically. The reason is that the reduction in the electrode width results in a reduction in the amount of charge transferred. The open circuit voltage for the 0.2 mm electrode width is only around half of that of 1.0 mm. The reduction in electrode width increases the number of electrode pairs covered by the slider at the same excitation amplitude. The output frequency of VI-TENG increases as the width becomes smaller. Then the width of the electrode is selected to be 0.5 mm as a compromise of signal amplitude and frequency. The output frequency also increases in this test as the excitation amplitude goes up, which verifies the feasibility of characterizing vibration amplitude with VI-TENG’s output frequency.

#### 3.1.3. Performance Experiments

**Figure 5:**
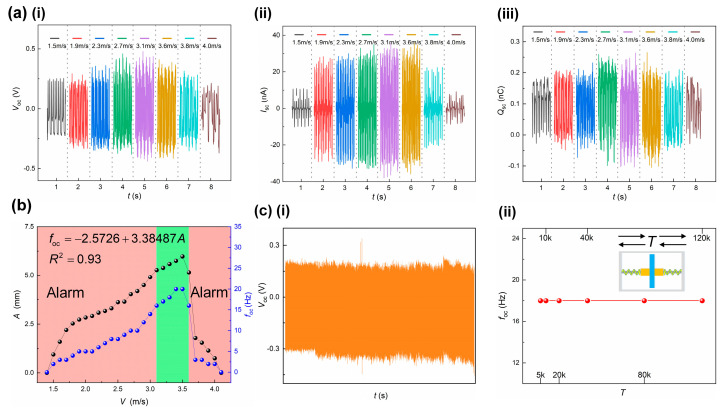
Output performance and durability test. (**a**) The real-time output performance of AVM: (i) open circuit voltage, (ii) short circuit current and (iii) amount of charge transferred; (**b**) the vibration amplitude and output frequency at different gas velocities; (**c**) durability performance: output voltage and frequency at 120,000 vibration repetitions: (i) open circuit voltage, (ii) frequency.

With the optimized parameters of vortex vibration and VI-TENG, the output performance of the AVM is tested in terms of gas velocity response and durability. Figure 5a demonstrates the real-time output signals of the open circuit voltage VOC, short circuit current ISC and amount of charge transferred QSC at different gas velocities. Figure 5b illustrates the gas velocity response of the vortex vibration amplitude and output frequency. The functional relationship between vortex vibration amplitude and output frequency shows it is feasible to characterize the amplitude in terms of frequency, and the correlation coefficient being 0.93, the supplementary data are presented in the Support Information. The frequency shows quite similar trend to the amplitude of vortex vibration as the gas velocity varies. In the meantime, the output frequencies at both the upper and lower threshold of gas velocity are 16 Hz, below which leakage alarm could be triggered. The durability test is conducted on a vibration exciter. The vibration amplitude is set to 5.5 mm which is the equivalent vibration amplitude of 3.3 m/s gas velocity, falling in the normal working range. The vibration repeats 120,000 cycles (Figure 5c). The output VOC has no obvious electrical signal attenuation. The output frequency is quite stable during the whole test. Using output frequency as the indicator of gas velocity, the AVM demonstrates very good durability performance.

### 3.2. Demonstration and Application

**Figure 6:**
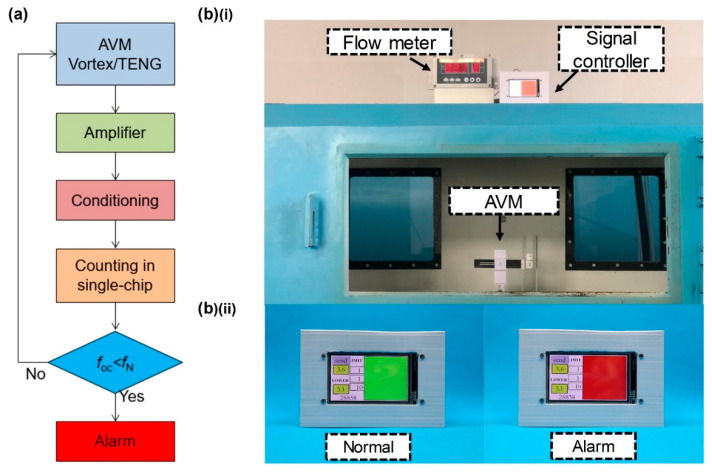
Demonstration of AVM application: (**a**) signal processing of the AVM; (**b**) test setup and the controller: (i) arrangement of test equipment and (ii) the situation when the controller displays normal or alarm.

To verify the performance of AVM, gas leakage alarm test is conducted. The wind tunnel was adopted to represent the ventilation pipeline and a flow meter was used to display the real-time gas velocity. A controller was designed to measure the output frequency of the AVM and trigger alarm upon gas leakage. Some details of the controller are shown in Supporting Information. It consists of an electrometer level amplifier, a signal conditioning circuit, a single-chip and a touch screen. An indicating square displays green for normal velocity and red for alarm. The signal processing flow chart is shown in Figure 6a. The AVM is fixed in the wind tunnel to monitor the gas velocity. The AVM controller is placed next to the flow meter to facilitate observation, as shown in Figure 6b. The two conditions: that the gas velocity goes from normal range to above the upper threshold and to below the lower threshold, were tested to verify the alarm capability. The demonstration result is shown in Videos S1 and S2. The indicating square turns red as the pipeline flow rate goes beyond the upper and lower threshold. The AVM could generate alarm for gas leakage.

## 4. Conclusions

An air velocity monitor for coal mine ventilation based on vortex-induced vibration and triboelectric nanogenerator is proposed in this study. The air velocity is represented by the amplitude of the vortex-induced vibration. The vortex-induced triboelectric nanogenerator further transforms the amplitude signal into signal frequency which has good durability. Parameters of the vortex-induced vibration and vortex-induced triboelectric nanogenerator are optimized with experiments. The relationship between the output frequency and gas velocity is also tested. Conclusions of this study include:

- Vortex-induced vibration could be used for air velocity monitor. The length of the beam and mass of the slider should be optimized to match the upper and lower threshold. Therefore, 42.5 mm and 0.4 g are the optimized values in this study.
- Vortex-induced triboelectric nanogenerator is applicable to transform vibration amplitudes into signal frequency, the correlation coefficient of linear regression is 0.93.
- The AVM is effective in generating and alarm for air velocity monitor. The output frequency goes below 16 Hz as the gas velocity goes above the upper threshold 3.6 m/s or below the lower threshold 3.1 m/s.

## Data Availability

Not applicable.

## Supplementary Materials

The following supporting information can be downloaded at: https://www.mdpi.com/article/10.3390/s22134832/s1, Table S1: Table of nomenclature, Figure S1: Details of the vibration experiment, Figure S2: The relationship between vibration amplitude and VI-TENG frequency; Video S1: The situation when flow velocity increased, Video S2: The situation when flow velocity decreased.
