# Supplementary material for: An Air Velocity Monitor for Coal Mine Ventilation Based on Vortex-Induced Triboelectric Nanogenerator
Source: Sensors (Basel). 2022 Jun 26;22(13):4832. doi: 10.3390/s22134832 (PMC9268754; doi:10.3390/s22134832)
Supplement: Supplementary file 1 [file sensors-22-04832-s001.zip › Support Information.pdf]

Supporting Information
An Air Velocity Monitor for Coal Mine Ventilation Based on Vortex-induced Triboelectric Nanogenerator

Guocheng Shen [1], Jijie Ma [1,2], Yili Hu[1], Jianping Li [1], Tinghai Cheng [3,*], Jianming Wen[1,2,*]

The Institute of Precision Machinery and Smart Structure, College of Engineering, Zhejiang Normal University, Yinbin Street 688, Jinhua, 321004, China
[2] Key Laboratory of Intelligent Operation and Maintenance Technology & Equipment for Urban Rail Transit of Zhejiang Province, Zhejiang Normal University, Yinbin Street 688, Jinhua, 321004, China
[3] Beijing Institute of Nanoenergy and Nanosystems, Chinese Academy of Sciences, Beijing 101400, China
* Correspondence: chengtinghai@binn.cas.cn, wjming@zjnu.cn

Table S1. Nomenclature.

Symbol	Description
$L(\mathrm{mm})$	length of the beam
$m(g)$	mass of the slider
$V(\mathrm{m}/s)$	gas velocity
$V_{\mathrm{U}}(\mathrm{m/s})$	upper velocity of gas
$V_{\mathrm{L}}(\mathrm{m/s})$	lower velocity of gas
$A(\mathrm{mm})$	vibration amplitude of the bluff body
$f(\mathrm{Hz})$	vibration frequency of the bluff body
$D(\mathrm{mm})$	width of the electrodes
$d(\mathrm{mm})$	space between electrodes
$f_{oc}(\mathrm{Hz})$	output frequency of the electrical signal

The vibration system consists of vibration control system, Power amplifier and Shaker [Figure S1(a)], which excited AVM perform simple harmonic motion to simulate vortex-induced vibration. The input frequency is 5.4Hz (the natural frequency of the AVM), the input acceleration is from 0.02g to 0.07g with step of 0.01g. The output vibration amplitude and output frequency of the AVM are obtained [Figure S1(b)], and the accelerations of 0.02g, 0.04g and 0.07g are selected (covering the vortex-induced vibration amplitude in the operating speed range) for optimization experiments. Figure S1(c) shows the real-time vibration signal.

The output frequency of VI-TENG are fitted with the independent of vibration amplitude [Fig. S2]. The fitting equation is shown in S1, where a=-2.5726 and b=3.384.

$$f_{oc}=\mathrm{a}+\mathrm{b}*A \qquad \text{(S1)}$$

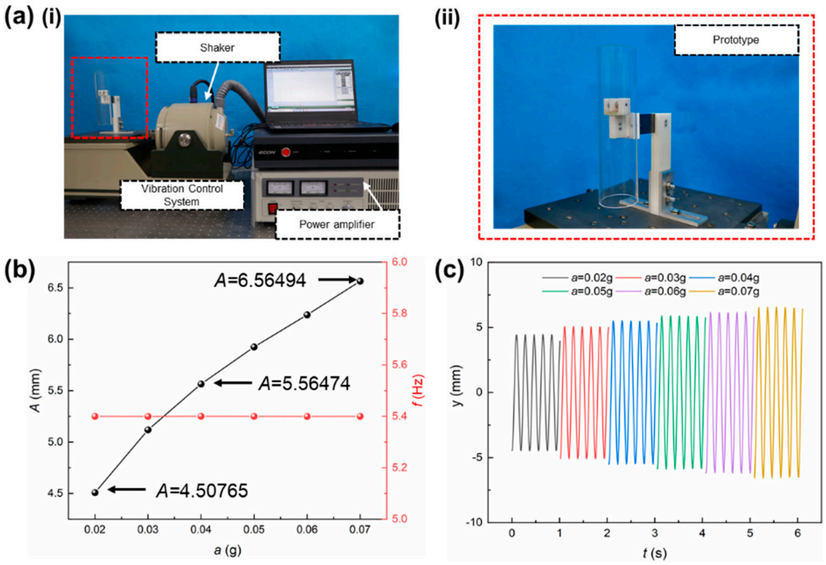

Figure S1. Details of the vibration experiment. (a) Composition of the vibration system; (a) The output vibration amplitude and frequency of the AVM; (c) Real-time vibration signals.

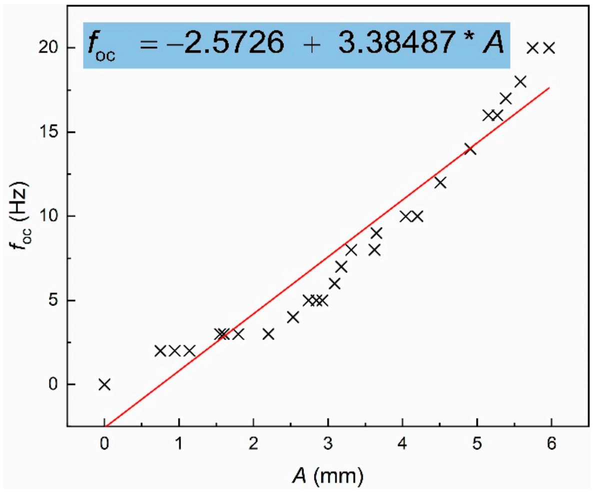

Figure S2. The relationship between vibration amplitude and VI-TENG frequency.

The operation of the controller includes the participation of hardware and software. The input signal is amplified by E-metcr operation amplifier OPA128. A singlechip is adopted to read the signal and calculate the frequency. The program reads and filters the amplified signal, then identifies the cycle of the filtered signal and calculates the frequency.
